# Complete Genome Sequence of a Novel Subgenotype of Porcine Reproductive and Respiratory Syndrome Virus Strain JX/CH/2016, Isolated in Jiangxi, China

**DOI:** 10.1128/genomeA.00980-17

**Published:** 2017-09-14

**Authors:** Fanfan Zhang, Deping Song, Yu Ye, Nannan Guo, Min Zhang, Hao Li, Dan Lei, Wang Gong, Suxian Luo, Dongyan Huang, Yuxin Tang

**Affiliations:** Key Laboratory for Animal Health of Jiangxi Province, Nanchang, Jiangxi, China, and Department of Preventive Veterinary Medicine, College of Animal Science and Technology, Jiangxi Agricultural University, Nanchang, Jiangxi, China

## Abstract

We sequenced the complete genome of porcine reproductive and respiratory syndrome virus (PRRSV) strain JX/CH/2016. Phylogenetic analysis based on the sequences of the open reading frame 5 (ORF5) gene revealed that this strain belongs to subgenotype IV. This is the first report of the complete genome sequence of PRRSV-IV.

## GENOME ANNOUNCEMENT

Porcine reproductive and respiratory syndrome virus (PRRSV), the causative agent of porcine reproductive and respiratory syndrome (PRRS), is a small, enveloped, positive-sense, single-stranded RNA virus that belongs to the *Arteriviridae* family (1, 2). According to its genetic and antigenic characteristics, PRRSV has been divided into two major genotypes, the European type (Lelystad virus [LV] is the representative prototype) and the North American type (VR-2332 virus is the prototype) (3–5). The epidemiology of PRRSV in China is complex. The first reported PRRSV strain, CH-1a, was identified in 1996. Highly pathogenic PRRSV (HP-PRRSV) strains, such as JXA1, first occurred in 2006 and since then have remained the predominant strains in China (3, 6). In addition, genovariations of PRRSVs have been frequently observed. Recently, several provinces in China reported outbreaks of NADC30-like PRRSVs with genovariations existing in the open reading frame 5 (ORF5) region of the genome of PRRSVs (2, 3, 7–11). Herein, we report the full-length genome sequence and molecular characteristics of a novel subgenotype of the PRRSV strain JX/CH/2016 isolated in Jiangxi, China.

JX/CH/2016 was isolated from a lung sample of a nursing pig that had been vaccinated with a commercial HP-PRRSV vaccine in a farrow-to-finish farm in Jiangxi Province, China. To determine the complete genome of JX/CH/2016, 16 pairs of primers with overlapping were designed and applied to amplify the complete genome by reverse transcription-PCR (RT-PCR). The 5′ untranslated region (UTR) and 3′ UTR of JX/CH/2016 were determined by use of a Smarter rapid amplification of cDNA ends (RACE) amplification kit (Clontech, USA). Sequences of all fragments of JX/CH/2016 amplified were assembled and annotated using DNAStar Lasergene software v 7.1.

Excluding the 3′ poly(A) tail, the full-length genomic sequence of JX/CH/2016 comprises 15,226 nucleotides (nt). The genome of JX/CH/2016 had a typical genome organization of *Arterivirus* and was arranged in the order of the 5′ UTR (nt 1 to 188), ORF1a (nt 189 to 7520), ORF1b (nt 7508 to 11890), ORF2a (nt 11892 to 12662), ORF2b (nt 11897 to 12118), ORF3 (nt 12515 to 13279), ORF4 (nt 13060 to 13596), ORF5 (nt 13607 to 14209), ORF6 (nt 14194 to 14718), ORF7 (nt 14708 to 14879), and the 3′ UTR (nt 14880 to 15226). Genetic homologous analyses showed that the complete genome of JX/CH/2016 shared 58.4%, 88.7%, 91%, 86.1%, and 82.2% nucleotide identities with the Lelystad virus, CH-1a, JXA1, VR2332, and NADC 30, respectively. In addition, an analysis based on the GP5 gene of PRRSV revealed that JX/CH/2016 had 85.0% amino acid (aa) identity with the representative HP-PRRSV strain of JXA1 and 59.9% aa identity with the representative European PRRSV strain of Lelystad. Phylogenetic analysis indicated that JX/CH/2016 formed a single branch, subgenotype IV (PRRSV-IV), a novel subgenotype of PRRSVs.

In conclusion, we determined the full-length genome sequence of PRRSV strain JX/CH/2016. To our knowledge, this is the first report on PRRSV-IV. Our results will provide insights into the genetics of a PRRSV, as well as information on the epidemiology and evolution of PRRSVs in China.

### Accession number(s).

The complete genome sequence of the JX/CH/2016 strain is available in GenBank under the accession number KY495780.
